# Putting a Porcelain Facing on a Living Honey-Combed Incisor

**Published:** 1896-12

**Authors:** P. Headridge


### Abstracts and Translations.

### PUTTING A PORCELAIN FACING ON A LIVING HONEY-COMBED INCISOR.

BY P. HEADRIDGE, L.D.S.I.

THE object of this operation is to improve the appearance of a honey-combed tooth without destroying the nerve.

In the case demonstrated the tooth was shortened as much as possible without cutting too near the pulp, as the tooth was found to be too frail towards the cutting edge to serve as a support for the porcelain face. (In cases where it is possible it is better to leave the palatal surface of the natural tooth entire, as a better support is given to the porcelain face, and there is then less danger of fracture from pressure of the bite.) The labial surface was ground flat down to a line or so below the level of the gum. During the grinding the tooth was isolated by the rubber dam and a stream of ethyl chloride was kept playing upon it, thus rendering the operation nearly painless. The natural tooth thus prepared presented a flat labial surface and a flat surface at the cutting edge. A plaster impression of the tooth was taken and cast with fusible metal. A good thick vulcanite tooth (Ash's make) of the right shade was selected, and the pins cut off and the tooth fitted to the metal model. The shoulder of the vulcanite tooth was fitted to the flat surface at the cutting edge of the natural tooth, the fine fitting being accomplished in the mouth. Two holes were next drilled in the natural tooth on the labial surface, one on either side of the pulp-chamber, and in doing this the demonstrator pointed out that they must not be drilled through to the palatal surface. A very narrow strip of platinum plate (No. 4 or 5), less than one-sixteenth of an inch wide, was fitted so that an end dips into each hole. The mineral face was then smeared with wax in order to ascertain where the groove was to be cut for the platinum to be fused into.

After the grove had been cut the bent platinum pin was held in its place in the groove by a trace of wax. The mineral face, with the pin waxed to it, was next placed on the natural tooth to obtain the exact position in the mouth. The face was then removed with the platinum still waxed to it and a low fusing body placed round the platinum, so that when the body fused it filled up the groove and so fused the platinum pin securely in its place. The face, when baked was cemented with the natural tooth with an osteoplastic. Special care must be taken that there is no undue pressure from the bite, and it is also advisable to leave the cement several days until it is thoroughly hardened. The edges may then be finished off and polished, and the operation thus completed.—*Journ. British Dental Association.*

